# Removal of Scale-Forming Ions and Oil Traces from Oil Field Produced Water Using Graphene Oxide/Polyethersulfone and TiO_2_ Nanoribbons/Polyethersulfone Nanofiltration Membranes

**DOI:** 10.3390/polym14132572

**Published:** 2022-06-24

**Authors:** Tarek Ashraf, Nada Alfryyan, Mervat Nasr, Sayed A. Ahmed, Mohamed Shaban

**Affiliations:** 1 Chemistry Department, Faculty of Science, Beni-Suef University, Beni-Suef 62514, Egypt; ch.tarekash@gmail.com (T.A.); mervatnasr94@gmail.com (M.N.); skader_70@yahoo.com (S.A.A.); 2 Nanophotonics and Applications (NPA) Lab, Physics Department, Faculty of Science, Beni-Suef University, Beni-Suef 62514, Egypt; 3 Department of Physics, College of Sciences, Princess Nourah Bint Abdulrahman University, P.O. Box 84428, Riyadh 11671, Saudi Arabia; 4 Department of Physics, Faculty of Science, Islamic University of Madinah, Almadinah Almonawara 42351, Saudi Arabia

**Keywords:** GO/PES nanofiltration membrane, TNR/PES nanofiltration membrane, scale-forming ions removal, oil traces removal

## Abstract

Treatment of produced water in oil fields has become a tough challenge for oil producers. Nanofiltration, a promising method for water treatment, has been proposed as a solution. The phase inversion technique was used for the synthesis of nanofiltration membranes of polyethersulfone embedded with graphene oxide nanoparticles and polyethersulfone embedded with titanium nanoribbons. As a realistic situation, water samples taken from the oil field were filtered using synthetic membranes at an operating pressure of 0.3 MPa. Physiochemical properties such as water flux, membrane morphology, flux recovery ratio, pore size and hydrophilicity were investigated. Additionally, filtration efficiency for removal of constituent ions, oil traces in water removal, and fouling tendency were evaluated. The constituent ions of produced water act as the scaling agent which threatens the blocking of the reservoir bores of the disposal wells. Adding graphene oxide (GO) and titanium nanoribbons (TNR) to polyethersulfone (PES) enhanced filtration efficiency, water flux, and anti-fouling properties while also boosting hydrophilicity and porosity. The PES-0.7GO membrane has the best filtering performance, followed by the PES-0.7TNR and pure-PES membranes, with chloride salt rejection rates of 81%, 78%, and 35%; oil rejection rates of 88%, 85%, and 71%; and water fluxes of 85, 82, and 42.5 kg/m^2^ h, respectively. Because of its higher hydrophilicity and physicochemical qualities, the PES-0.7GO membrane outperformed the PES-0.7TNR membrane. Nanofiltration membranes embedded with nanomaterial described in this work revealed encouraging long-term performance for oil-in-water trace separation and scaling agent removal.

## 1. Introduction

Water scarcity and sustainable water use have long been a global problem, thus industrial wastewater treatment has become a top priority. Produced water, which is a frequent expression in the oil and gas industry for wastewater generated as a byproduct in the oil and gas industry, has been a challenge for many years because there is a legal obligation to dispose of it without harming the environment. The problem is posed by a large amount of produced water, as American oil fields typically produce ten barrels of water for every barrel of oil produced, although the global ratio is more commonly 3:1 [1]. Oil fields can dispose of produced water in a variety of ways, including discharging it in the sea in the case of offshore sites after treatment to comply with environmental regulations that are tightening up, while traditional techniques are insufficient to treat produced water to comply with the new regulations. As a result of new laws, oil in water must be less than 25 ppm to meet European standards [2]. Re-injecting it into specified water disposal wells is another option. In this case, treatment of produced wastewater is also an important first step in reducing scaling agents like calcium, magnesium, strontium, and barium, which are present in the form of CaSO_4_ (anhydrite, gypsum), BaSO_4_ (barite), SrSO_4_ (celestite), and CaCO_3_ (calcium carbonate), to minimize the tendency of scale formation. Scale formation causes damage to the well’s reservoir by decreasing permeability [3]. When produced water with varying chemical concentrations of sulfates, calcium, magnesium, strontium, and barium ions are mixed, or when temperature and pH are altered, scale development can occur [4], resulting in a decrease in reservoir permeability, which implies the well can no longer receive more water. Another issue is oil traces that remain suspended in produced water after the majority of the oil has been separated from the water-oil (*w/o*) emulsion in the oil field using methods such as heating, adding chemicals (demulsifier), and increasing settling time in tanks, but these methods are ineffective in removing oil traces with droplet sizes smaller than 10 μm [5]. Oil droplets might clump together with scale and sand in the reservoir’s pores, reducing permeability. Scale development and greasy water both raise the expense of wells intervention, such as increasing permeability or drilling a nearby water disposal well. This problem can be solved by using nanofiltration(NF)to reduce scaling ion concentrations to acceptable levels and remove oil droplets. Using filtration membranes in produced water treatment has many advantages such as high permeate quality, lesser space required, ease of operation and no chemicals needed, while disadvantages of this method are membrane fouling, low flux, and short membrane lifetime. Polymers such as polysulphone (PS), polyether sulphone (PES), polyacrylonitrile (PAN), polyvinylidene fluoride (PVDF), and cellulose acetate (CA) are widely used in the fabrication of nanofiltration and possess high efficiencies of oil removal from produced water with privileges of low cost and disadvantages of low flux and fouling tendency [6]. Many trials were performed to enhance produced water treatment using nanofiltration, as Chitosan/Pluronic F108/Polyethersulfone Membrane which hadoil rejection in the range of 80–90% with water flux 170 (L·m^−2^h·^−1^) [7] and polyethersulfone/TiO_2_ nanomaterial ultrafiltration membrane had oil rejection with an efficiency of 92% [8].

Nanofiltration (NF) membranes have gone a long way since their discovery in the late 1980s with qualities that fall somewhere between ultrafiltration (UF) and reverse osmosis (RO)filtration. The pore size of NF membranes ranges from 1 to 10 nm, which correlates to a molecular weight cut-off of 300–500 Da [9]. Because of the dissociation of surface functional groups or the adsorption of charged solutes, NF membranes in contact with an aqueous solution are also mildly charged. Polymeric NF membranes, for example, have ionizable groups such as carboxylate and sulfonate, which result in a charged surface in the presence of a feed solution. NF membranes are distinguished by their low rejection of monovalent ions, a strong rejection of multivalent ions, and increased flux when compared to RO membranes.NF membranes have high selectivity, high chemical stability, high permeability, and can work under high pressures, and, in some cases, high temperatures. Because of their mechanical, thermal, and chemical stability, polysulfone (PS) and polyethersulfone (PES) are appealing materials for NF membranes, however, their hydrophobic nature is their main disadvantage that contributes to the membrane fouling [10]. Therefore, nanomaterials such as GO and TiO_2_ nanostructures were introduced to improve PES membrane performance. Quanling et al. studied the effect of adding graphene oxide to PES to increase salt rejection and water permeability [11]. Nadiret al. studied the antifouling and self-cleaning properties of GO/PES membranes [12]. Arcadio et al. investigated PES-TiO_2_ nanofiltration membranes for enhancing permeability and dye rejection [13]. Guiping et al. studied the design and characterization of PES/TiO_2_ membranes [14]. Moreover, PES/TiO_2_ hollow fiber membranes were investigated by Silvia et al. for water desalination [15]. Abdulaziz et al. used graphene-based nanocomposite membranes for oil traces removal from produced water with oil rejection of 99.9% and water flux of 91.3 kg m^−2^ h^−1^ bar^−1^ [16]. Mishra et al. used poly(1,4 phenylene ether sulphone) with PVP and TiO_2_ to synthesize the UF membrane and employed it for filtration of synthetic oily feed with94.7% oil rejection, 88% salt rejection and 35 kg m^−2^ h^−1^ water flux [17]. Yuliwati et al. had an oil rejection of 98% and water flux of 82.5 kg m^−2^ h^−1^ using a PVDF-TiO_2_ ultrafiltration membrane [18].

Despite some of the mentioned research utilizing GO or TiO_2_ in water treatment [10,11,12,13,14,15], however, there is no detailed report focused on the use of NF membranes in the filtration of a realistic produced water sample from an oil field, with a focus on both scale-forming ions and oil content, as both must be treated before being discharged into the environment or disposed into injection wells (to maintain permeability without reservoir bores blockage). Alammar et al. [16] and Mishra et al. [17] employed alternative membrane construction parameters and synthetic oily water rather than genuine oil field samples. Additionally, the salt rejection was investigated in terms of TDS (total dissolved salts), but there was no detailed study of the most significant scale-forming ions (chloride, calcium, magnesium, and barium), which pose a high danger of clogging reservoir pores in water injection wells. Therefore, water samples from oil fields must be used to reflect the true composition of produced water. The produced water includes a variety of compounds such as corrosion inhibitors, biocide, scale inhibitors, oxygen scavengers, H_2_S scavengers, and other chemicals used during drilling and production operations, dissolved and dispersed oil compounds (hydrocarbons), corrosion products, and sulfate reduced bacteria (SRB). These chemicals have the potential to diminish or fail the filtering process and can wreak havoc on filtering efficiency and fouling results. Therefore, this work aims to introduce a category of low-cost nanofillers by incorporating titanium nanoribbons (TNR) and graphene oxide (GO) into the PES membrane to solve for the first time the problem of the contaminated water produced during the oil production industry and to get rid of the oil traces, calcium, chloride, magnesium, and barium ions. This technique can reduce the blocking of the oil reservoir pores and prevent the decrease of reservoir permeability. Therefore, in this study, TNRs and GO nanomaterials are fabricated by hydrothermal and modified Hummer methods and incorporated into PES membranes to increase filtration efficiency and overcome low water flux and membrane fouling. The results of filtration of produced water samples collected from oil fields containing calcium, chloride, magnesium and bromide ions with oil traces are comparatively studied using pure PES, PES-GO, and PES-TNR in terms of filtration efficiency, water flux, and fouling tendency. In other words, the filtration of realistic water samples from oil fields has been investigated in this study to prove the usefulness of this method to be directed to manufacturing and play a role in protecting the environment and solving the produced water problem in the oil production industry. Moreover, the advantages of the manufactured membranes become obvious by comparing salt and oil rejection results with a commercial membrane.

## 2. Experimental Details

### 2.1. Materials

PES (polyethersulfone, MW = 58,000 g mol^−1^) from BASF company (Ludwigshafen, Germany) and DMF N(N-dimethylformamide) from Sigma-Aldrich (Darmstadt, Germany), titanium dioxide from LobaChemie (Mumbai, India), graphite powder, O-phosphoric acid, sulfuric acid, sodium hydroxide, hydrochloric acid from Sigma-Aldrich (Darmstadt, Germany), and potassium permanganate and hydrogen peroxide were obtained from PIOCHEM (Cairo, Egypt).

### 2.2. Preparation of GO Nanostructure

Graphene oxide nanoparticles were fabricated by a modified Hummer’s method by using 1 g of graphite powder added to a mixture of 120 mL 96%wt H_2_SO_4_ and 14 mL 85%wt H_3_PO_4_ in an ice bath, 6 g of KMnO_4_ powder added slowly over 1 h on a magnetic stirrer, then the solution was left for 1 day at 50 °C in a water bath. The combination of acid and potassium permanganate will result in the intercalation of acid in the graphite layer and oxidation into graphene oxide flakes. Following that, 800 mL deionized water and 10 mL H_2_O_2_ were added drop by drop alternately in an ice bath to reduce the residual of the KMnO_4_, resulting in a lot of air bubbles and a reddish yellow color, and the solution was left to settle for 24 h before being filtered. Finally, the precipitate is washed with deionized water till the solution becomes neutral (pH = 7) and then dried at 60 °C to obtain GO powder [19].

### 2.3. Synthesis of TNRs

Synthesis of titanium nanoribbons was carried out using a hydrothermal technique by adding 4 g of titanium dioxide to 500 mL sodium hydroxide with a concentration of 10 M, then, using a magnetic stirrer, the mixture was stirred for half an hour untiled homogenous solution appeared. Then, the mixture was poured into a 1 L Teflon-lined stainless-steel autoclave. The autoclave was placed in the oven for 24 h at 170 °C and after cooling the resultant powder was rinsed with 0.1 M hydrochloric acid and distilled water. The obtained white powders were left to dry at 80 °C for 24 h before calcination at 450 °C for 2 h [20].

### 2.4. Synthesis of PES-GO and PES-TNR Membranes

Membranes were synthesized by the phase inversion method [21] by adding nanomaterial powder TNR in the case of PES-TNR membranes and GO in the case of PES-GO membranes to 50 mL DMF gradually. The quantity of nanostructured powder was added according to the reported weight% in Table 1. The powder was added during the stirring for ~3 h followed by the addition of DMF to reach a 100 mL mixture. The mixtures were kept under stirring for over 24 h until they formed a homogenous emulsion. Then 17.5 g of PES powder was added gradually to the emulsion over an ultrasonic vibrator at 45 °C. Then, stirring was continued for 12 h. After that, the emulsion was cast on a clean glass plate using a thin-film casting device with 120 μm thickness. Finally, the cast membranes were immersed in distilled water at 45 °C to be hardened then removed and dried in the air [22].

### 2.5. Memberane Characterization Techniques

#### 2.5.1. Hydrophilicity and Porosity

Membrane hydrophilicity before and after adding nanomaterials was determined by measuring the contact angle using the sessile method by contact angle analyzer (KRÜSS, DSA100, Hamburg, Germany) for the synthesized membranes.

The porosity of the synthesized membranes, which is pores volume divided by total membrane volume, was determined by immersing the membrane in distilled water. Then, excess water was removed using filter paper and the weight (W_1_) of the membrane was measured. Then, the membrane was left to dry for 4 h at 85 °C and the weight of the dry membrane (W_2_) was measured [22]. The membrane porosity can be estimated by Equation (1):

(1) $$\varepsilon_{m}=\frac{\left(w_{1}-w_{2}\right)/\rho_{w}}{\frac{w_{1}-w_{2}}{\rho_{w}}+w_{2}/\rho_{p}}$$

where ε_m_ is porosity (%), W_1_ and W_2_ are the weight of the wet and dry membranes(in grams), $\rho_{w}$ is the density of pure water (0.999 g cm^−3^) and $\rho_{p}$ is the density of PES (1.37 g cm^−3^).

The mean pore radius was then determined using the filtration velocity method according to the Guerout-Elford-Ferry Equation (2):

(2) $$r_{m}=\sqrt{\frac{8\left(2.9-1.75\varepsilon_{m}\right)\eta Ql}{\varepsilon_{m}\;A∆P}}$$

where η is the water viscosity (8.9 × 10^−4^ Pas), Q is the volume of the permeate pure water per unit time (m^−3^·s), l is the membrane thickness (m) and ∆P  is the operation pressure 0.3 MPa.

#### 2.5.2. Morphologies and Structures

Field emission-scanning electron microscopy (FE-SEM, Model quanta 250 field emission gun, FEI, Shinagawa, Japan) and a transmission electron microscope (TEM JEOL, Model 2010, Tokyo, Japan) were used for the characterization of membrane morphologies. The TNR’s and GO’s crystallographic structures were investigated using X-ray diffraction (Philips X’Pert Pro MRD, Malvern Panalytical Ltd., Malvern, UK) by an EMPYREAN diffractometer with Cu Kα radiation operated at 30 mA and 40 kV in the 2θ range 5–80°.

### 2.6. Membrane Performance

#### 2.6.1. Filtration Process

The filtration procedure was carried out using a pressurized system as shown in Figure S1 (Supplementary data) that included a pressurizing pump that sucked the produced water sample and pumped it to the membrane cell at a discharge pressure of 0.3 MPa. Many bench-scale experiments of filtration membranes with variable applied pressure have employed a pressurizing pump [8,23,24,25]. For dye and salt separation, Fang et al. employed a peristaltic pump with a low-pressure feed of 0.2 MPa for nanofiltration employing the PES-iron ion complex [26]. For water treatment, Hudaib et al. employed a ceramic TiO_2_ nanofiltration membrane with a feed pressure of 0.2 MPa [27]. For wastewater treatment, Amin et al. utilized 0.27 MPa on PES/GO nanoribbons and PES/GO nanosheets [28]. Filtration was performed three times using a circular flat sheet specimen with a surface area of 28.2 cm^2^ and a feed pressure of 0.3 MPa. The filtration system has a three-opening flat sheet membrane module for feeding, concentrating, and permeating [29]. Using a high-pressure pump, the feed was continually delivered to the membrane module from a closed feeding tank (50 L). The permeated water was then collected from the membrane modules downstream. The findings of manufactured membranes were compared to those of a commercial nanofiltration membrane, NTR-7450. Nitto-Denko of Switzerland provided the NTR-7450 membrane. The top layer of the NTR-7450 membrane is sulfonated polyethersulfone (SPES), which is covered with an ultrathin polymeric composition layer, according to the manufacturer. The top barrier’s semi-permeability is determined by its properties (solvent flux and solute rejection). SPES contains aromatic rings, oxygen, and sulfonic groups [30].

#### 2.6.2. Water Flux

The water flux of the synthesized pure-PES, PES-TNR and PES-GO membranes was investigated by using deionized water as feed water and observing the quantity of permeate every 5 min, where there was a decline in permeate quantity for the first hour, then the permeability rate increased and remained constant. The quantity of water was measured when the rate became constant [31]. Water flux (*J_w_*) was determined using the following equation.

(3) $$J_{w}=\frac{M}{A\cdot \Delta t}$$

where *J_w_* is pure water flux (kg/m^2^ h), *M* is mass of permeate (kg), *A* is the effective area of membrane (m^2^) and Δt is the filtration time (h).

#### 2.6.3. Filtration Efficiency

Filtration was performed on a sample of produced water taken from an oil field in the West Bakr Company in Egypt’s western desert, with chemical concentrations as listed in Table 2.

Filtration efficiencies of produced water from its constituent ions were investigated by comparing the concentration of chloride, calcium, magnesium, and barium before and after filtration. After one hour of commencing the filtering process, permeate water samples were taken when the water flux became steady. Ion concentrations were determined according to ASTM D-4327 for chloride anions and ASTM D-6919 for calcium, magnesium, and barium cations, respectively, using ion chromatography. The Dionex ICS 1100 was used, which was outfitted with high-capacity columns (AS9 and CS12) for anions and cations [32,33,34]. The efficiency of produced water filtration from oil in water content was investigated by measuring oil in water content using the Eracheck analyzer device according to the ASTM D-7678 standard method [35]. Analyses were carried out by adding 5 cyclohexane to 45 mL of tested sample and aggressively shaking the mixture, then allowing it to rest until two phases of water at the bottom and the hydrocarbon-Cyclohexane mixture at the top were separated. The top layer was then withdrawn with a syringe and combined with 0.1 g sodium sulfate as a drying agent to remove any water traces. Finally, the extracted hydrocarbon portion was submitted to an infrared device with wave number 1370–1380 cm^−1^ and multiplied results with the concentration factor. The concentration factor is determined by dividing the sample volume by the solvent volume (concentration Factor = 9). Salt and oil rejection (R%) is determined using the following equation:

(4) $$R\%\;=\frac{\mathrm{Co}-C}{\mathrm{Co}}\times 100$$

where Co is feed ion concentration or oil content (as indicated in Table 2), and C is ion concentration or oil content in the permeate.

#### 2.6.4. Fouling Tendency

Fouling tendencies of produced water were investigated by a dynamic method using produced water with the same composition and oil in water concentration as a fouling agent. The first filtration using synthesized membranes using deionized water was performed for one hour at a discharge pressure of 0.3 MPa and water flux was recorded (J_w1_), then produced water was used to perform filtration at the same parameters of time and pressure. The membranes were then washed with 1 M NaOH at 0.3 MPa and water for 30 min. The cleaning chemical NaOH was chosen because of its quick cleaning outcomes and overall cleaning effectiveness [36]. Filtration used pure water while recording related water flux (J_W2_), the flux recovery ratio (F_RR_) [37] was calculated using the following equation:

F_RR_% = J_w2_/J_w1_ × 100 (5)

#### 2.6.5. Alkaline Resistance and Evaluation of Successive Filtration

To evaluate the alkaline resistance of the membranes, PES-0.7 GO and PES-0.7 TNR membranes were immersed in 1 M NaOH for three days, and water flux and salt rejection were compared to previous results.

Double successive filtrations were carried out to see if it could result in increased filtration efficiency. In a comparative study, the salt rejection and oil rejection for single filtration and double-successive filtration were assessed.

## 3. Results and Discussion

### 3.1. Hydrophilicity of Synthesized Membranes

**Figure 1:**
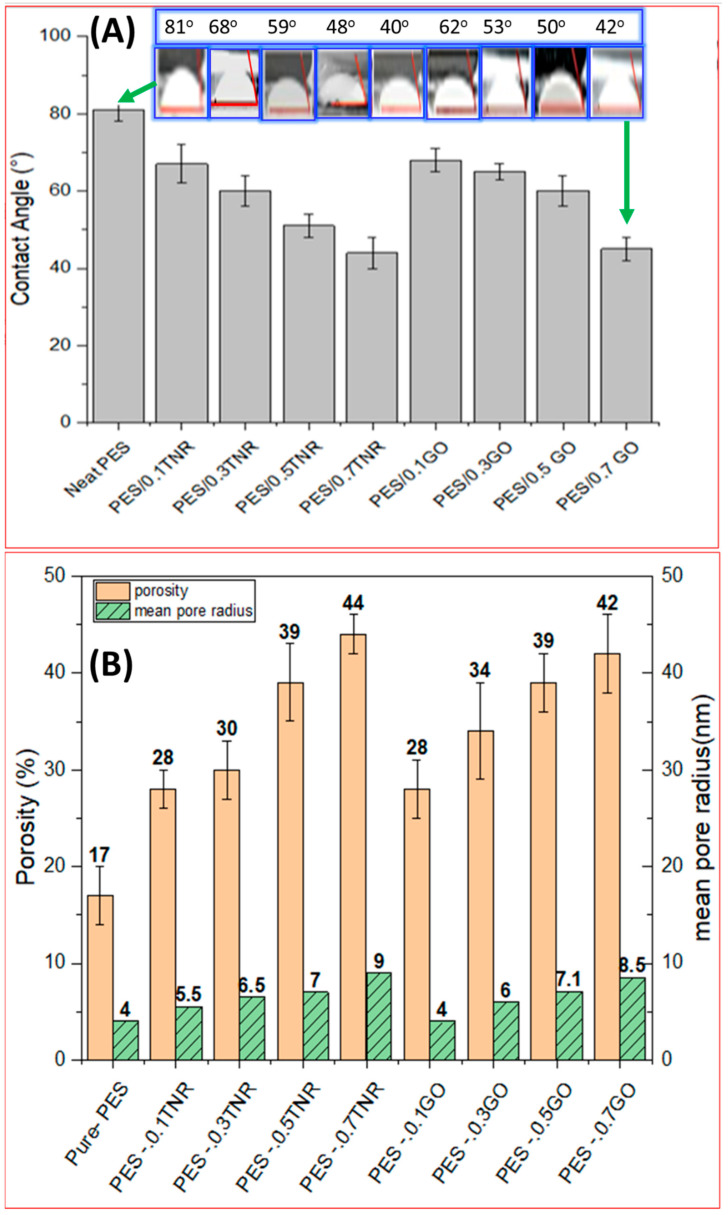
(**A**) Contact angle and (**B**) porosity and mean pore radius of the synthesized membranes.

The sessile drop method [38] is applied to determine the contact angles of the synthesized membranes by measuring the angle between a deionized water droplet and a dry membrane surface immediately after dropping the droplet with a ‘I’-shaped needle on the membrane surface. The process is repeated five times and the average of the readings is reported [39]. Figure 1A shows that increasing the nanomaterial percentage in the membrane results in lower contact angle readings, indicating that increasing the nanomaterial percentage increases hydrophilicity [40,41]. Pure-PES membranes have the largest contact angle and are the least hydrophilic. The hydrophilic nature of the TNR [42] and GO nanomaterial is primarily responsible for this considerable shift. The hydrophilicity of GO results from the presence of C=O, C-O-C, and C-OH functional groups [43]. During the NIPS (Non-solvent induced phase separation) process, nanomaterial spontaneously moves to the polymer/water interface to reduce the interface energy. After the membranes have been fully formed, the majority of the nanomaterial particles migrate to the membrane surface and stabilize, enhancing hydrophilicity.

**Figure 2:**
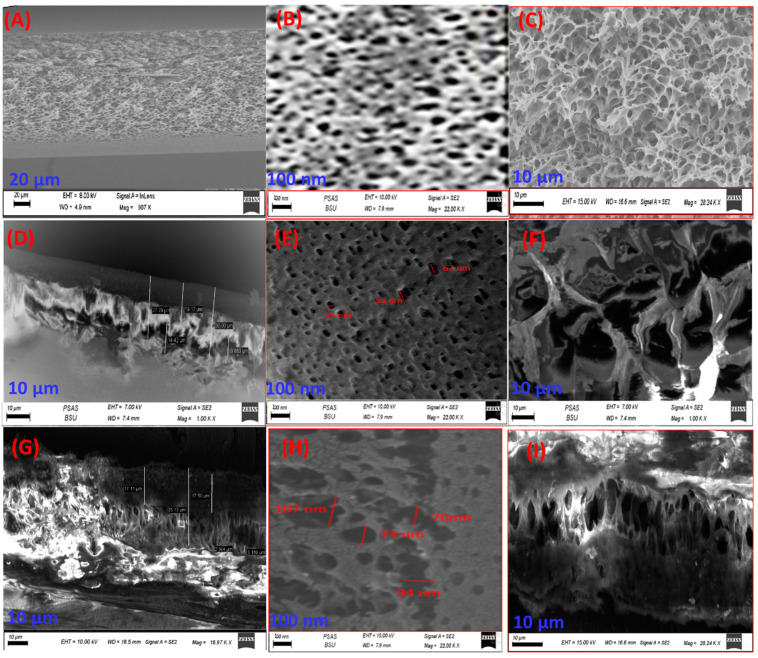
Cross-sectional SEM micrographs for (**A**–**C**) pure PES, (**D**–**F**) PES-0.7GO, and (**G**–**I**) PES-0.7TNR membranes.

Figure 1B shows that increasing the ratio of nanomaterial increases the porosity of the membranes. The microstructure of generated membranes is influenced by the thermodynamic and kinetic components of the phase inversion process. Nanomaterial was used to increase porosity. The thermodynamic effect of adding nanomaterial resulted in uniform dispersion and the hydrophilic character of the added nanomaterial increased hydrophilicity and accelerated non-solvent adsorption, increasing the polymer film’s thermodynamic instability. During the phase inversion process, quick demixing resulted in the production of membranes with a larger porosity. During non-solvent induced phase separation (NIPS), the high affinity of the solvent to cover embedded nanomaterial compared to polymer chains results in the creation of membranes with a larger porosity [44]. The membrane pore size is affected by the phase transition velocity of water–solvent during phase inversion. The mean pore size is estimated using Equation (2) and presented in Figure 1B for all studied membranes. The mean pore size of PES-GO and PES-TNR membranes increased as the amount of GO and TNR added increased, as shown in Figure 2B, which is due to the hydrophilicity properties of GO and TNR, which is compatible with the contact angle and porosity results. This is also consistent with the findings of Nurul et al. [45], who found that additive loadings increased the hydrophilicity and surface porosity of membranes. The PES-GO and PES-TNR membranes developed in this work had a reduced contact angle, higher porosity, and larger mean pore size, which resulted in superior hydrophilic characteristics.

### 3.2. Membrane Morphology

Figure 2A–C shows a cross-sectional and top view SEM image of the pure-PES membrane matrix with micro pits of near-identical sizes spread throughout. These SEM images illustrate the spongy-like structure of the pure-PES membrane. The SEM image in Figure 2C shows an asymmetric membrane with top and bottom porous surfaces. The cross-sectional SEM images in Figure 2D,F for PES-0.7GO indicate a denser and less porous top layer of the membrane, although the formation of macro voids was present in the sub-layer. Macro void lengths ranged from 8 to 14 µm. Figure 2E depicts the top morphology of the PES-GO membrane. The number of pores increased, and their diameters decreased when GO was introduced [45]. Figure 2G,I shows cross-sectional SEM images for the PES-0.7TNR membrane with macro voids ranging from 12 to 18 µm. Figure 2H shows the top layer of the PES-TNR membrane, whereas some of these pores exhibit coalescence. The insertion of TNRs results in the growth of lateral, larger pores at the top layer and macro voids in the sub-layer [25]. The overall thickness ranges from 80 to 100 µm for both PES-GO and PES-TNR membranes.

The hydrophilic character of the added GO and TNR nanomaterial, which promotes solvent and non-solvent mass transfer rates, is credited with the creation of nanopores and macro voids [46,47,48]. According to SEM micrographs, graphene oxide nanoparticles were efficiently dispersed in the polymer matrix and did not show significant aggregation on the membrane surface, which may be attributed to the graphene oxide carbon-based structure [46]. Anna Rabajczyk et al. [47,48] demonstrated that dispersing TNR or GO in DMF (solvent) while stirring prevents nanoparticle agglomeration.

**Figure 3:**
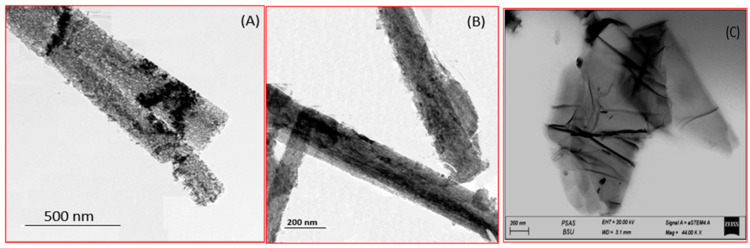
TEM image of (**A**,**B**) TNR and (**C**) GO nanomaterials.

Transmission electron microscopy (TEM) has long been recognized as a helpful method for describing the morphology of GO and TNR. TEM images, Figure 3A,B, of the calcinated TNR illustrate the influence of the calcination temperature on the surface of the produced nanoribbons. The conversion of hydrogen titanate to TiO_2_-B NRs resulted in the formation of pores with diameters ranging from 2 to 10 nm [49]. These pores are formed because the interlayered OH groups escape the structure [50,51]. Figure 3C illustrates the graphene oxide plates as flat surfaces. The effective synthesizing of the GO nanostructure is confirmed by the TEM image in Figure 3C. By oxidizing graphene extensively, the graphitic stack’s flakes were chemically exfoliated into mono- and multilayer sheets [52,53].

### 3.3. Structural Properties

**Figure 4:**
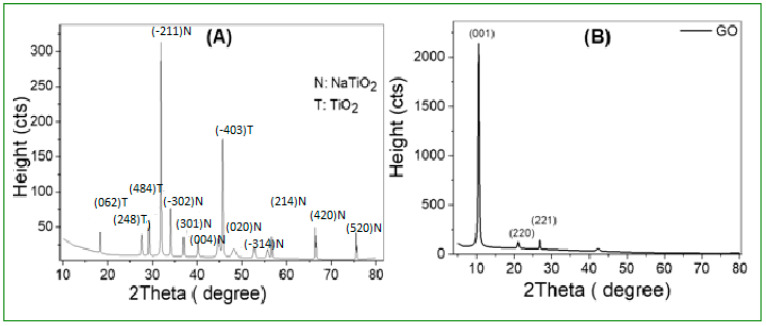
XRD charts of (**A**)TiO_2_ nanoribbons and (**B**) GO nanostructure.

Figure 4 illustrates the XRD charts of TiO_2_ nanoribbons and GO nanoparticles. For TiO_2_ nanoribbons, Figure 4A, monoclinic NaTiO_2_ and cubic TiO_2_ crystalline phases are observed from XRD charts. The peaks of TiO_2_ are detected at 2theta of 18.3°, 27.6°, 29.2°, and 45.8° indicating the presence of a cubic TiO_2_ phase along the (062), (248), (484), and (−403) orientations (card No. 96-413-0133) [54]. The peaks of NaTiO_2_ are detected at 2theta of 31.9°, 34.2°, 35.4°, 40.2°, 46.3°, 47.7°, 52.7°, 55.7°, 56.8°, 66.2°, and 75.4°indicating the presence of a monoclinic NaTiO_2_ phase along the (−211), (301), (−302), (004), (401), (020), (−314), (214), (015), (420), and (520) orientations (card No. 96-231-0332) [55]. However, for TiO_2_, the (−403) peak is the most intense, while for NaTiO_2_, the (−211) peak is the most intense.

Figure 4B shows the tetragonal GO crystalline phase in GO nanoparticles as seen on the XRD chart. The presence of a tetragonal GO phase along the (001), (220), (221), and (322) orientations is indicated by the presence of a GO peak at 2theta of 10.5°, 21°, 26.7°, and 42.3° (card No. 96-590-0025) [56], the (10.5°) peak is the most intense. This could be attributed to the fluctuation of equilibrium surface concentration in the direction of minimizing surface energy or setting the segregation energy near zero.

Using values of W (full width at half maximum) for XRD peaks, the average crystallite size of TiO_2_, NaTiO_2_, and GO is estimated using Scherrer’s equation:

(6) $$Ds=\frac{0.94\lambda }{\mathrm{Wcos}\;\theta }$$

where *Ds*, θ, and λ are the crystallite size, Bragg’s angle, and X-ray wavelength, respectively [57,58]. Texture coefficients (TCs) and dislocation densities [59] are also calculated. Table 3 shows the structural parameters that were computed. The average Ds values are 65.6, 66.5, and 23.6 nm for TiO_2_, NaTiO_2,_ and GO, respectively. In general, the smaller crystallite size can be attributed to the formation of additional nucleating centers as the nucleation energy barrier is reduced [60]. The highest texture coefficient (TC) values were 1.42, 4.56, and 3.66, indicating that TiO_2_, NaTiO_2,_ and GO grew best along (484), (−211), and (001), respectively. Moreover, the smallest density of crystal defects, particularly dislocation density, is detected for TiO_2_, NaTiO_2_ and GO, respectively, along (062), (−211), and (221), with values of 0.12 × 10^−3^, 0.24 × 10^−3^, and 0.61 × 10^−3^ dis/nm^2^.

### 3.4. Membrane Performance

#### 3.4.1. Evaluation of Filtration Efficiency

**Figure 5:**
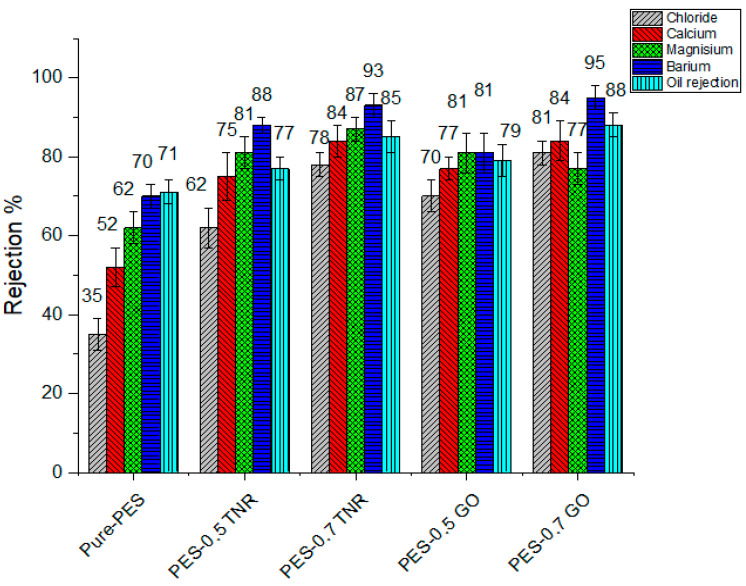
Salt and oil rejection of pure-PES, PES-TNR, PES-GO and NTR-7450 membranes.

For this investigation, ion concentration at feed and permeate water were measured. The permeated water was subsequently tested by ion chromatography in the same way, yielding the findings shown in Table 4. Figure 5 shows the salt rejection of the various membranes with close values, with the maximum efficiency forPES-0.7GO, followed by PES-0.7TNR, and the lowest efficiency for pure PES. PES with a higher nanomaterial ratio has a higher salt rejection, which can be interpreted as higher hydrophilicity. This finding is in line with that of Zylla et al., who discovered that decreasing nanofiltration membrane contact angles enhanced salt rejection [61]. Sulfonic and ether groups were commonly found in the PES layer on top of the NF membrane. In addition, the skin layer has mean radius pores of 6.5–11 nm. As a result, a complicated process including electrostatic contact and steric hindrance was responsible for the separation of solutes by NF membranes. When comparing findings to commercial NTR-7450 membrane salt rejection, it seems that the NTR-7450 membrane has somewhat higher salt rejection than pure PES and slightly lower salt rejection than PES-TNR and PES-GO. The hydrophobic character of the commercial membrane can be linked to the reduction in salt rejection at NTR-7450 [54]. The commercial membrane is covered with an ultrathin polymeric layer, which results in the membrane being hydrophobic despite the presence of sulfonic acid groups. The results show that membranes may be used at high concentrations of generated water. GO-toluidine/PES membranes yielded encouraging findings for Zhongzhen et al. [62]. Increased hydrophilicity causes the diffusion rate of water molecules to rise, whereas salt ions are trapped in pores.

#### 3.4.2. Efficiency of Oil Removal

Table 5 shows the removal of oil from water utilizing synthesized membranes. The concentration of oil in water at feed water was 120 ppm. The oil rejection increased from 71% to 88% by increasing the ratio of GO content from 0% to 0.7% and increased to 85% by increasing the TNR to 0.7%, i.e., the highest oil in water rejection (88%) was reported for PES-0.7 GO versus 85% for PES-0.7 TNR and71%for the pure PES, which is considered the lowest reported value for our prepared membranes. This behavior is reversely correlated to the values of the contact angle. This suggests that the difference in filtering effectiveness is due to membrane hydrophilicity, which produces repulsion between hydrophilic membrane surfaces and hydrophobic oil droplets, allowing more water to enter the pores [63]. As observed in SEM images in Figure 2, the integration of 0.7% GO or 0.7%TNR in the PES membrane also increases pore density and porosity, which can contribute to the increase in oil rejection. Furthermore, as demonstrated in Table 5, our optimized membranes have a higher oil rejection than the commercial NTR-4750 membrane, which has 40% oil rejection. This is due to the commercial membrane’s hydrophobic nature.

#### 3.4.3. Water Flux and Flux Recovery Ratio

**Figure 6:**
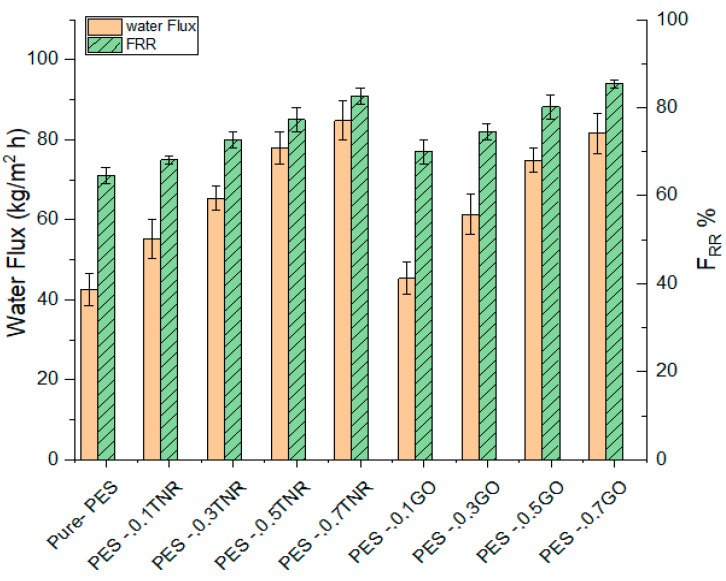
Pure water flux and the flux recovery ratio of synthesized pure-PES, PES-TNR, and PES-GO membranes.

Water flux of the synthesized membrane was evaluated by observing the permeate volume per unit time at a discharge pressure of 0.3 MPa. Figure 6 demonstrates that the PES-0.7GO membrane had a higher water flux than the PES-0.7TNR membrane, while the pure-PES membrane had the lowest water flux. This is owing to the PES-0.7GO membrane’s higher hydrophilicity than the PES-0.7TNR membrane, while pure PES has the lowest hydrophilicity [40,64]. The ability of water molecules to permeate across the selective layer of the membrane is increased by increasing the hydrophilicity. It might also be attributed to the inclusion of nanostructures, which results in the creation of macro voids and an increase in porosity and mean pore radius. The increase in porosity and mean pore radius after introducing nanomaterial is consistent with the results of water flux [65]. The findings of employing GO as a nanofiller material for nanofiltration membranes are consistent with the reported data by Hao et al. [25] and Amin et al. [61]. TiO_2_ nanomaterial, which is utilized in nanofiltration, also improves the water flux [66,67,68].

The fouling tendency of produced water was explored utilizing a flux recovery ratio (FRR) based on pure water flux before (Jw_1_) and after (Jw_2_) using synthesized membranes in produced water filtration. The higher FRR value indicates a higher antifouling tendency to get lower fouling. When comparing the antifouling tendency of pure-PES, PES-TNR, and PES-GO membranes, PES-0.7GO outperforms PES-0.7TNR, while pure PES has the lowest antifouling tendency. The hydrophilic interactions between the membrane surface and feed solution constituents increase electrostatic forces, resulting in improved antifouling. One of the most common methods for developing antifouling membrane surfaces is to increase the surface hydrophilicity, for example, by adding hydrophilic monomers or nanoparticles [66].

#### 3.4.4. Alkaline Resistance of Membranes

**Figure 7:**
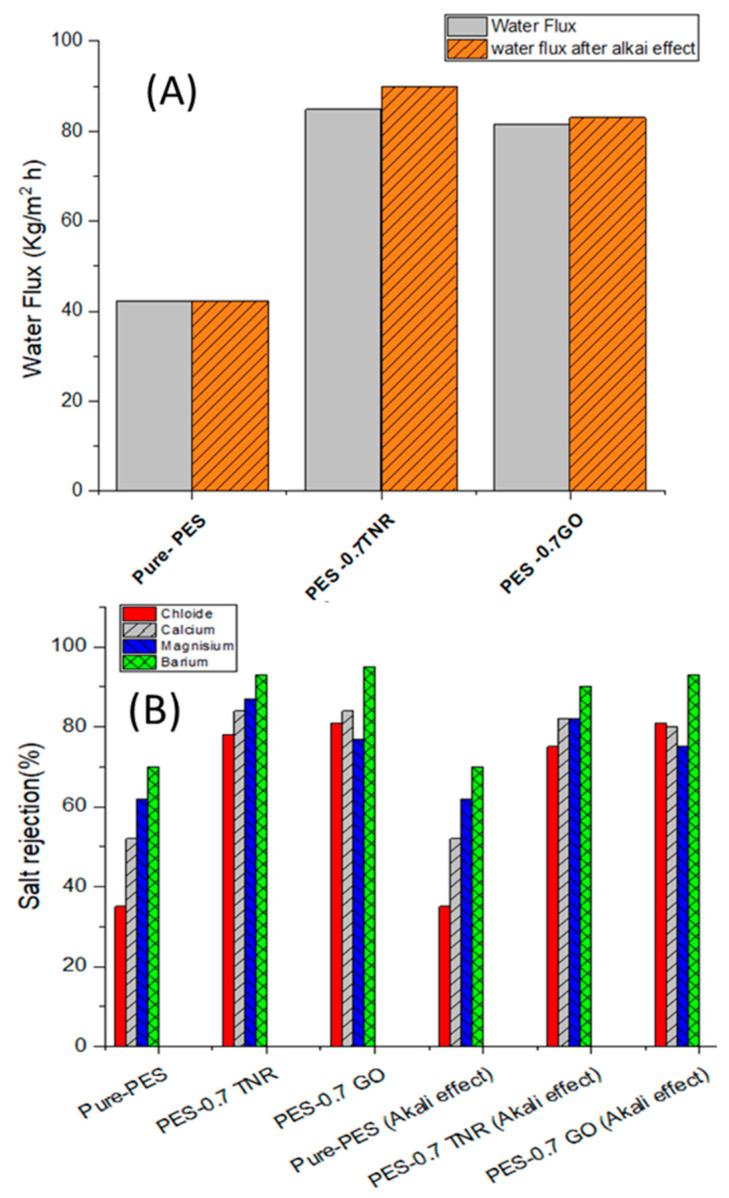
(**A**) Water flux and (**B**) salt rejection of synthesized pure-PES, PES-TNR, and PES-GO membranes before and after NaOH immersion for three days.

Only a few commercially available NF membranes, notably PES-based membranes, can tolerate high caustic conditions. The salt rejection and water flux stabilities in an alkaline environment were used to estimate the membranes’ alkaline resistance [69,70]. Both PES–0.7-GO and PES–0.7TNR demonstrated consistent salt rejection and water flux after three days of immersion in 1 M NaOH solution, as illustrated in Figure 7A,B. Both PES–0.7GO and PES–0.7TNR may be used as alkali-resistant NF membranes with increased filtering performance, according to this discovery. Due to the presence of aromatic groups, alternating sulphonyl (SO_2_) groups, and ether (-O-) linkages, PES has great thermal stability, a high glass transition temperature, and strong structural and chemical stability [71,72]. These findings are in line with those of Mehwish et al., who discovered that TiO_2_ NP can improve the chemical stability of PES membranes [66]. Yeh et al. have shown the remarkable stability of GO membranes in water [67]. The successful use of 1 M NaOH as a cleaning agent with a high FRR demonstrates membrane resilience to cleaning. The chemical stability of PES, which is attributable to the sulfonyl and ether groups, contributes to cleaning resistance.

#### 3.4.5. Evaluation of Successive Filtration

**Figure 8:**
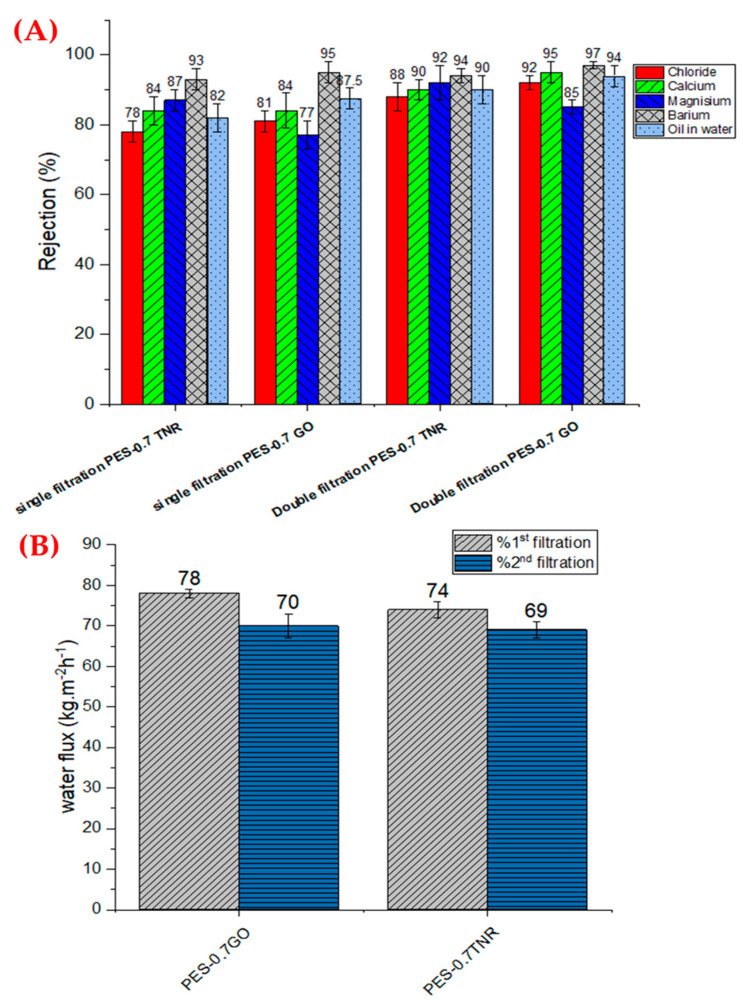
(**A**) Salt rejection% and (**B**) water flux using PES-0.7 TNR and PES-0.7 GO in a double filtration process.

The antifouling capabilities of PES-GO and PES-NR membranes, as well as the possibility of enhancing salt and oil rejection, were investigated further using a dynamic cyclic filtering experiment for generated water samples. Figure 8A demonstrates that in the second filtering cycle, the total rejection of ions and oil increased. Figure 8B shows how the fouling impact of produced water lowers water flux from 78 to 70 kg·m^−2^ h^−1^ for PES-0.7GO and 74 to 69 kg·m^−2^·h^−1^ for PES-0.7TNR during the second filtering cycle. The high flux recovery ratios of PES-0.7GO (89.7%) and PES-0.7TNR (93.2%) are demonstrated by the minor decrease in water flux. The limited decrease in water flux, salt rejection, and oil rejection refers to a very limited leaching effect for the used nanomaterials. The findings are in line with those of Li et al., who discovered that nanomaterial leaching from PES-TiO_2_ membranes was low for the first five hours, then stopped, whereas leaching was only strong at PES membranes substantially laden with nanomaterial [73]. Additionally, Cheng et al. observed that employing a GO-PES membrane grafted with polyampholyte hydrogel results in very low graphene oxide leaching, with less than 2% graphene oxide leached from the membrane [74]. This finding encourages businesses to use membranes to treat generated water in oil fields in order to comply with environmental regulations.

## 4. Conclusions

Nanofiltration membranes made of PES, PES-GO, and PES-TNR were created using the phase inversion approach. Membranes were used to filter generated water samples taken from an oil field in order to create a realistic atmosphere. The addition of nanomaterials increased membrane performance in several ways. Membranes’ physiochemical characteristics were studied. The addition of TNR or GO to the PES matrix causes macro voids to form on the bottom surface of the membrane in both PES-TNR and PES-GO. When the weight of the embedded nanostructure is increased, the contact angle decreases from 81°for pure PES to 40° and 42° for PES-0.7GOand PES-0.7 TNR, respectively. Porosity rose from 17% for pure PES to 44% for PES-0.7TNR and 42% for PES-0.7GO, respectively, while water flux increased from 42.5 kg/m^2^ h to 85 and 82 kg/m^2^ h. By increasing the weight percent of integrated nanomaterial, chloride, calcium, magnesium, and barium concentrations were reduced while water flux was increased. The PES-0.7GO membrane has the highest filtering efficiency, followed by the PES-0.7TNR and pure-PES membrane, with chloride salt rejection of 81%, 78%, and 35%, respectively. Furthermore, increased hydrophilicity is the main cause of increased oil rejection using the membranes of PES-0.7TNR (88%) and PES-0.7GO (85%) over the pure-PES membrane (71%) and commercial NTR-7450 (40%) due to repulsion forces between hydrophobic oil traces and the hydrophilic nature of nanostructure-incorporated membrane surfaces. Fouling tendency decreased with the addition of nanomaterial, with the lowest fouling tendency at the PES-0.7GO membrane, followed by the PES-0.7TNR membrane, and the greatest fouling tendency at the pure-PES membrane, which may be ascribed to fouling mitigation through enhanced hydrophilicity. Cleaning membranes with NaOH was tried before measuring flux recovery ratio, and the FRR was found to be quite high. Membrane resistance to alkali after three immersions demonstrated the membrane’s great resistance to alkaline media. Overall, the findings support the use of membranes in the oilfield to treat generated water.

## Figures and Tables

**Table 1:** The concentration of nanomaterial in synthesized membranes.

Membrane	PES wt%	Nanomaterials WT%	DMF wt%
PUREPES	17.50	-	82.50
PES/0.1GO	17.50	0.10	82.40
PES/0.3GO	17.50	0.30	82.20
PES/0.5GO	17.50	0.50	82.00
PES/0.7GO	17.50	0.70	81.80
PES/0.1TNR	17.50	0.10	82.40
PES/0.3TNR	17.50	0.30	82.20
PES/0.5TNR	17.50	0.50	82.00
PES/0.7TNR	17.50	0.70	81.80

**Table 2:** Produced water constitutes concentrations.

Constitute Ions	Concentration (ppm)
Chloride	10,220
Calcium	1391
Magnesium	273
Barium	70
Oil in water	120

**Table 3:** The structural parameters of TiO_2_, NaTiO_2_, and GO.

Compound Name	Peaks	TiO_2_	NaTiO_2_	GO
Crystal System		Cubic	Monoclinic	Tetragonal
XRD peak position(2θ°)	(a)	18.3	31.9	10.5
	(b)	27.6	34	21.1
	(c)	29.2	45.6	26.7
Miller indices (hkl)	(a)	(062)	(−211)	(001)
	(b)	(248)	(−302)	(220)
	(c)	(484)	(020)	(221)
Relative integrated intensity (I/Io) %	(a)	9.3	100	212
	(b)	9	57.5	18
	(c)	16.6	23	5.6
Mean crystallites size (D (nm))	(a)	91.8	64.7	23.1
	(b)	40.6	65	19.5
	(c)	64.3	25.2	40.5
Texture coefficient (TC)	(a)	0.80	4.56	3.66
	(b)	0.77	1.05	0.11
	(c)	1.42	2.62	0.18
Dislocation density (dis/nm^2^) × 10^−3^	(a)	0.12	0.24	1.87
	(b)	0.61	0.24	2.63
	(c)	0.24	1.57	0.61

**Table 4:** Permeate concentration after filtration using synthesized membranes and related filtration efficiencies.

	Chloride	Calcium	Magnesium	Barium
Feed Conc. (mg/L)	10,220	1391	273	70
Permeate Conc. pure-PES membrane (mg/L)	6643	668	104	21
Salt rejection	35%	52%	62%	70%
Permeate Conc. PES-0.5TNR membrane(mg/L)	3884	348	52	8
Salt rejection	62%	75%	81%	88%
Permeate Conc. PES-0.7TNR membrane (mg/L)	2248	223	35	5
Salt rejection	78%	84%	87%	93%
Permeate Conc. PES-0.5GO membrane (mg/L)	3066	320	52	13
Salt rejection	70%	77%	81%	81%
Permeate Conc. PES-0.7GO membrane (mg/L)	1942	181	63	4
Salt rejection	81%	87%	77%	95%
NTR-7450 membrane	5928	598	96	15
Salt rejection	42%	57%	65%	78%

**Table 5:** Oil in water removal efficiency using synthesized membranes.

Oil in Water at Feed Water	120 ppm	Oil Rejection
PurePES	35 ppm	71% ± 3
PES-0.1GO	33 ppm	73% ± 2
PES-0.3GO	29 ppm	76% ± 3
PES-0.5GO	25 ppm	79% ± 4
PES-0.7GO	15 ppm	88% ± 3
PES-0.1TNR	40 ppm	67% ± 2
PES-0.3TNR	30 ppm	75% ± 2
PES-0.5TNR	28 ppm	77% ± 3
PES-0.7TNR	18 ppm	85% ± 4
NTR7450	72 ppm	40% ± 2

## Data Availability

Not applicable.

## Supplementary Materials

The following supporting information can be downloaded at: https://www.mdpi.com/article/10.3390/polym14132572/s1, Figure S1. Schematic of filtration system.

## References

[1] Liu Z.M., Jin Y.Q., Yuan G.Q., Law M.J. (2013). The Treatment and Disposal of Produced Water from Onshore Oilfields. Appl. Mech. Mater..

[2] MKhedr M.G. (2014). Nanofiltration of oil field-produced water for reinjection and optimum protection of oil formation. Des. Water Treat..

[3] Bin Merdha A.B., Yassi A.A.M. (2007). Scale Formation in Oil Reservoir During Water Injection at High-Salinity Formation Water. J. Appl. Sci..

[4] Stalker R., Collins I.R., Gordon M.G. (2003). The Impact of Chemical Incompatibilities in Commingled Fluids on the Efficiency of a Produced Water Reinjection System: A North Sea Example, Houston, Texas. International Symposium on Oilfield Chemistry.

[5] Zhu Y., Wang D., Jiang L., Jin J. (2014). Recent progress in developing advanced membranes for emulsified oil/water separation. NPG Asia Mater..

[6] Baek Y., Kang J., Theato P., Yoon J. (2012). Measuring hydrophilicity of RO membranes by contact angles via sessile drop and captive bubble method: A comparative study. Desalination.

[7] Hamzah N., Rohani R., Hassan A.R., Sharifuddin S.S., Isa M.H.M. (2018). Development of chitosan/pluronic F108/polyethersulfone (PES) nanofiltration (NF) membrane for oily wastewater treatment. AIP Conference Proceedings.

[8] Hosseini S.S., Fakharian Torbati S., Alaei Shahmirzadi M.A., Tavangar T., Fabrication J. (2018). Characterization, and performance evaluation of polyethersulfone/TiO_2_ nanocomposite ultrafiltration membranes for produced water treatment. Polym. Adv. Technol..

[9] Mohammad A., Teow Y., Ang W.L., Chung Y.T., Oatley-Radcliffe D., Hilal N. (2015). Nanofiltration membranes review: Recent advances and future prospects. Desalination.

[10] Van der Bruggen B., Braeken L., Vandecasteele C. (2002). Flux decline in nanofiltration due to adsorption of organic compounds. Sep. Purif. Technol..

[11] Xie Q., Zhang S., Xiao Z., Hu X., Hong Z., Yi R., Shao W., Wang Q. (2017). Preparation and characterization of novel alkali-resistant nanofiltration membranes with enhanced permeation and antifouling properties: The effects of functionalized graphene nanosheets. RSC Adv..

[12] Dizge N., Gonuldas H., Ozay Y., Ates H., Ocakoglu K., Harputlu E., Yildirimcan S., Unyayar A. (2016). Synthesis and performance of antifouling and self-cleaning polyethersulfone/graphene oxide composite membrane functionalized with photoactive semiconductor catalyst. Water Sci. Technol..

[13] Sotto A., Boromand A., Balta S., Darvishmanash S., Kim J., Van der Bruggen B. (2011). Nan-ofiltration membranes enhanced with TiO_2_ nanoparticles: A comprehensive study. Desalination Water Treat..

[14] Wu G., Gan S., Cui L., Xu Y. (2008). Preparation and characterization of PES/TiO_2_ composite membranes. Appl. Surf. Sci..

[15] Simone S., Galiano F., Faccini M., Boerrigter M.E., Chaumette C., Drioli E., Figoli A. (2017). Preparation and Characterization of Polymeric-Hybrid PES/TiO_2_ Hollow Fiber Membranes for Potential Applications in Water Treatment. Fibers.

[16] Alammar A., Park S.-H., Williams C.J., Derby B., Szekely G. (2020). Oil-in-water separation with graphene-based nanocomposite membranes for produced water treatment. J. Membr. Sci..

[17] Mishra S.B., Sachan S.B.S., Mishra P.K., Ramesh M. (2014). R ‘Preparation and characterisation of PPEES-TiO_2_ composite micro-porous UF membrane for oily water treatment. Procedia Mater. Sci..

[18] Yuliwati E., Ismail A. (2011). Effect of additives concentration on the surface properties and performance of PVDF ultrafiltration membranes for refinery produced wastewater treatment. Desalination.

[19] Kapitanova O.O., Panin G.N., Baranov A.N., Kang T.W. (2012). Synthesis and Properties of Graphene Oxide/Graphene 359 Nanostructures. J. Korean Phys. Soc..

[20] Li Q., Zhang J., Liu B., Li M., Liu R., Li X., Ma H., Yu S., Wang L., Zou Y. (2008). Synthesis of High-Density Nanocavities inside TiO_2_-B Nanoribbons and Their Enhanced Electrochemical Lithium Storage Properties. Inorg. Chem..

[21] Hołda A.K., Vankelecom I.F.J. (2015). Understanding and guiding the phase inversion process for synthesis of solvent resistant nanofiltration membranes. Appl. Polym. Sci..

[22] Shaban M., AbdAllah H., Said L., Hamdy H.S., Khalek A.A. (2014). Titanium dioxide nanotubes embedded mixed matrix PES membranes characterization and membrane performance. Chem. Eng. Res. Des..

[23] Cakmakci M., Baspinar A.B., Balaban U., Uyak V., Koyuncu I., Kinaci C. (2012). Comparison of nanofiltration and adsorption techniques to remove arsenic from drinking water. Desalination Water Treat..

[24] Mahvi A.H., Malakootian M., Fatehizadeh A., Ehrampoush M.H. (2011). Nitrate removal from aqueous solutions by nanofiltration. Desalination Water Treat..

[25] Junaidi N.F., Othman N.H., Shahruddin M.Z., Alias N.H., Marpani F., Lau W.J., Ismail A.F. (2020). Fabrication and characterization of graphene oxide–polyethersulfone (GO–PES) composite flat sheet and hollow fiber membranes for oil-water separation. J. Chem. Technol. Biotechnol..

[26] Fang X., Wei S., Liu S., Li R., Zhang Z., Liu Y., Zhang X., Lou M., Chen G., Li F. (2022). Metal-Coordinated Nanofiltration Membranes Constructed on Metal Ions Blended Support toward Enhanced Dye/Salt Separation and Antifouling Performances. Membranes.

[27] Hudaib B., Hajarat R., Liu Z. (2019). The Ceramic TiO_2_ Low-Pressure Nano-Filtration Membrane Separation Behavior for Single and Mixed Ion Salt Solutions. Jordanian J. Eng. Chem. Ind..

[28] Karkooti A., Yazdi A., Chen P., McGregor M., Nazemifard N., Sadrzadeh M. (2018). Development of advanced nanocomposite membranes using graphene nanoribbons and nanosheets for water treatment. J. Membr. Sci..

[29] Shaban M., Ashraf A.M., AbdAllah H., El-Salam H.M.A. (2018). Titanium dioxide nanoribbons/multi-walled carbon nanotube nanocomposite blended polyethersulfone membrane for brackish water desalination. Desalination.

[30] Her N., Amy G., Chung J., Yoon J., Yoon Y. (2008). Characterizing dissolved organic matter and evaluating associated nanofiltration membrane fouling. Chemosphere.

[31] Junaidi N.F.D., Khalil N.A., Jahari A.F., Shaari N.Z.K., Shahruddin M.Z., Alias N.H., Othman N.H. (2018). Effect of Graphene Oxide (GO) on the Surface Morphology & Hydrophilicity of Polyethersulfone (PES). IOP Conf. Ser. Mater. Sci. Eng..

[32] (2017). Standard Test Method for Determination of Dissolved Alkali and Alkaline Earth Cations and Ammonium in Water and Wastewater by Ion Chromatography.

[33] (2017). Standard Test Method for Anions in Water by Chemically Suppressed Ion Chromatography.

[34] Michalski R. (2006). Ion Chromatography as a Reference Method for Determination of Inorganic Ions in Water and Wastewater. Crit. Rev. Anal. Chem..

[35] Forrester S., Janik L., McLaughlin M., Soriano Disla J.M. (2013). An infrared spectroscopic test for total petroleum hydrocarbon (TPH) contamination in soils, Total Petroleum Hydrocarbon Concentration Prediction in Soils Using Diffuse Reflectance Infrared Spectroscopy. Soil Sci. Soc. Am. J..

[36] Zondervan E., Raffel B. (2007). Evaluation of different cleaning agents used for cleaning ultrafiltration membranes fouled by surface water. J. Membr. Sci..

[37] Marjani A., Nakhjiri A.T., Adimi M., Jirandehi H.F., Shirazian S. (2020). Effect of graphene oxide on modifying polyethersulfone membrane performance and its application in wastewater treatment. J. Sci. Rep..

[38] Zhang W., Wahlgren M., Sivik B. (1989). Membrane characterization by the contact angle technique II characterization of UF membranes and comparative between the captive bubble and the sessile drop as methods to obtain water contact angles. Desalination.

[39] Jepsen K.L., Bram M.V., Pedersen S., Yang Z. (2018). Membrane Fouling for Produced Water Treatment: A Review Study from a Process Control Perspective. Water.

[40] Fatin M., Ruslinda A.R., Arshad M.K.M., Hashim U., Norhafizah S., Farehanim M.A. Surface Functionalization of Multiwalled Carbon Nanotube for Biosensor Device Application. Proceedings of the 2014 IEEE International Conference on Semiconductor Electronics.

[41] Bolis V., Busco C., Ciarletta M., Distasi C., Erriquez J., Fenoglio I., Livraghi S., Morel S. (2012). Hydrophilic/hydrophobic features of TiO_2_ nanoparticles as a function of crystal phase, surface area and coating, in relation to their potential toxicity in peripheral nervous system. J. Colloid Interface Sci..

[42] Navaneethaiyer U., Mohan R., Lee J., Kim S.-J. (2012). Graphene oxide nanostructures modified multifunctional cotton fabrics. Appl. Nanosci..

[43] Qu P., Tang H., Zhang L.-P., Wang S. (2010). Polyether sulphone composite membrane blended with cellulose fibrils. Bioresources.

[44] Rahimi M., Zinadini S., Zinatizadeh A.A., Vatanpour V., Rajabi L., Rahimi Z. (2016). Hydrophilic goethite nanoparticle as a novel antifouling agent in fabrication of nanocomposite polyethersulfone membrane. Appl. Polym. Sci..

[45] Zinadini S., Zinatizadeh A.A., Rahimi M., Vatanpour V., Zangeneh H. (2014). Preparation of a novel antifouling mixed matrix PES membrane by embedding graphene oxide. J. Membr. Sci..

[46] Gholami N., Mahdavi H. (2018). Nanofiltration composite membranes of polyethersulfone and graphene oxide and sulfonated graphene oxide. Adv. Polym. Technol..

[47] Lin R., Hernandez B.V., Ge L., Zhu Z. (2018). Metal organic framework based mixed matrix membranes: An overview on filler/polymer interfaces. J. Mater. Chem. A.

[48] Umek P., Bittencourt C., Guttmann P., Gloter A., Škapin S.D., Arčon D. (2014). Mn^2+^ Substitutional Doping of TiO_2_ Nanoribbons: A Three-Step Approach. J. Phys. Chem..

[49] Bittencourt C., Rutar M., Umek P., Mrzel A., Vozel K., Arčon D., Henzler K., Krüger P., Guttmann P. (2015). Molecular Nitrogen in N-doped TiO_2_ Nanoribbons. RSC Adv..

[50] Zhang M., Jin Z., Zhang J., Guo X., Yang J., Li W., Wang X., Zhang Z. (2004). Effect of annealing temperature on morphology, structure and photocatalytic behaviour of nanotubed H_2_Ti_2_O4(OH)_2_. J. Mol. Catal. A Chem..

[51] Yuan Y.-G., Gurunathan S. (2017). Combination of graphene oxide–silver nanoparticle nanocomposites and cisplatin enhances apoptosis and autophagy in human cervical cancer cells. Int. J. Nanomed..

[52] Dave S.H., Gong C.C., Robertson A.W., Warner J.H., Grossman J.C. (2016). Chemistry and structure of graphene oxide via direct imaging. ACS Nano.

[53] Rabajczyk A., Zielecka M., Cygańczuk K., Pastuszka Ł., Jurecki L. (2021). Nanometals-Containing Polymeric Membranes for Purification Processes. Materials.

[54] Gao M.-Y., Wang F., Gu Z.-G., Zhang D.-X., Zhang L., Zhang J. (2016). Fullerene-Like Polyoxotitanium Cage with High Solution Stability. J. Am. Chem. Soc..

[55] Andersson S., Wadsley A.D. (1961). The crystal structure of Na_2_Ti_3_O_7_. Acta Crystallogr..

[56] Soomro S.A., Gul I.H., Naseer H., Marwat S., Mujahid M. (2019). Improved Performance of CuFe_2_O_4_/rGO Nanohybrid as an Anode Material for Lithium-ion Batteries Prepared Via Facile One-step Method. Curr. Nanosci..

[57] Shaban M., Zayed M., Hamdy H. (2017). Preparation and characterization of nanostructured ZnO thin films for self-cleaning Applications. RSC Adv..

[58] Shaban M., Mustafa M., El Sayed A.M. (2016). Structural, optical, and photocatalytic properties of the spray deposited nanoporousCdS thin films; influence of copper doping, annealing, and deposition parameters, Materials Science in Semiconductor Processing. Mater. Sci. Semicond. Processing.

[59] Shaban M., Ali M., Abdelhady K., Hamdy H. (2015). Al_2_O_3_ and Sn/Al_2_O_3_ nanowires: Fabrication and Characterization. Micro Nano Lett..

[60] Zayed M., Ahmed A.M., Shaban M. (2019). Synthesis and characterization of nanoporousZnO and Pt/ZnO thin films for dye degradation and water splitting applications. Int. J. Hydrogen Energy.

[61] Wang Z., Ma C., Xu C., Sinquefield S.A., Shofner M.L., Nair S. (2021). Graphene oxide nanofiltration membranes for desalination under realistic conditions. Nat. Sustain..

[62] Żyłła R., Foszpańczyk M., Kamińska I., Kudzin M., Balcerzak J., Ledakowicz S. (2022). Impact of Polymer Membrane Properties on the Removal of Pharmaceuticals. Memberanes.

[63] Kumar S., Nandi B.K., Guria C., Mandal A. (2017). Oil Removal from Produced Water by Ultrafiltration using Polysulfone Membrane. Braz. J. Chem. Eng..

[64] Mänttäri M., Pihlajamäki A., Nyström M. (2006). Effect of pH on hydrophilicity and charge and their effect on the filtration efficiency of NF membranes at different pH. J. Membr. Sci..

[65] Liang H., Zoua C., Tang W. (2021). Development of novel polyethersulfone mixed matrix membranes to enhance antifouling and sustainability: Treatment of oil sands produced water (OSPW). J. Taiwan Inst. Chem. Eng..

[66] Rabia M., Shaban M., Adel A., Abdel-Khaliek A.A. (2019). Effect of Plasmonic Au Nanoparticles on the Photoactivity of Polyaniline/Indium Tin Oxide Electrodes for Water Splitting. Environ. Prog. Sustain. Energy.

[67] Batool M., Shafeeq A., Haider B., Ahmad N. (2021). TiO_2_ Nanoparticle Filler-Based Mixed-Matrix PES/CA Nanofiltration Membranes for Enhanced Desalination. Membranes.

[68] Zangeneh H., Zinatizadeh A.A., Zinadini S., Feyzi M., Bahnemann D.W. (2018). A novel photocatalytic self-cleaning PES nanofiltration membrane incorporating triple metal-nonmetal doped TiO_2_ (K-B-N-TiO_2_) for post treatment of biologically treated palm oil mill effluent. React. Funct. Polym..

[69] Shalaby M.S., Naddeo V., Bore L., Abdallah H., Shaban A.M., Zarra T., Belgiorno V. (2018). Development of highly flux antifouling RO polyethersulfone membrane using compacted woven support. J. Membr. Sci. Res..

[70] Jeon S.I., Rajabzadeh S., Okamura R., Ishigami T., Hasegawa S., Kato N., Matsuyama H., Kobe University (2016). The Effect of Membrane Material and Surface Pore Size on the Fouling Properties of submerged membranes. Water.

[71] Tsehaye M.T., Velizarov S., Van der Bruggen B. (2018). Stability of polyethersulfone membranes to oxidative agents: A review. Polym. Degrad. Stab..

[72] Guan R., Zou H., Lu D., Gong C., Liu Y. (2005). Polyethersulfone sulfonated by chlorosulfonic acid and its membrane characteristics. Eur. Polym..

[73] Li X., Fang X., Pang R., Li J., Sun X., Shen J., Han W., Wang L. (2014). Self-assembly of TiO_2_ nanoparticles around the pores of PES ultrafiltration membrane for mitigating organic fouling. J. Membr. Sci..

[74] Zhang W., Cheng W., Ziemann E., Be’er A., Lu X., Elimelech M., Bernstein R. (2018). Functionalization of ultrafiltration membrane with polyampholyte hydrogel and graphene oxide to achieve dual antifouling and antibacterial propert. J. Membr. Sci..

