# Supplementary material for: Removal of Scale-Forming Ions and Oil Traces from Oil Field Produced Water Using Graphene Oxide/Polyethersulfone and TiO2 Nanoribbons/Polyethersulfone Nanofiltration Membranes
Source: Polymers (Basel). 2022 Jun 24;14(13):2572. doi: 10.3390/polym14132572 (PMC9269001; doi:10.3390/polym14132572)
Supplement: Supplementary file 1 [file polymers-14-02572-s001.zip › polymers-1753600-supplementary.pdf]

polymers

Supplementary

Removal of Scale-Forming Ions and Oil Traces from Oil Field Produced Water Using Graphene Oxide/Polyethersulfone and TiO_2 Nanoribbons/Polyethersulfone Nanofiltration Membranes

Tarek Ashraf [1,2], Nada Alfryyan [3,*], Mervat Nasr [1,2], Sayed A. Ahmed [1] and Mohamed Shaban [2,4,*]

1 Chemistry Department, Faculty of Science, Beni-Suef University, Beni-Suef62514, Egypt; ch.tarekash@gmail.com (T.A.); mervatnasr94@gmail.com (M.N.); skader_70@yahoo.com (S.A.A.)
2 Nanophotonics and Applications (NPA) Lab, Physics Department, Faculty of Science, Beni-Suef University, Beni-Suef 62514, Egypt, mssfadel@aucegypt.edu (M.S.)
3 Department of Physics, College of Sciences, Princess Nourah bint Abdulrahman University, P.O. Box 84428, Riyadh 11671, Saudi Arabia; naalfryyan@pnu.edu.sa
4 Department of Physics, Faculty of Science, Islamic University of Madinah, Al-Madinah Al-Munawarah42351, Saudi Arabia; mssfadel@aucegypt.edu (M.S.)
* Correspondence: naalfryyan@pnu.edu.sa (N.A.); mssfadel@aucegypt.edu (M.S.)

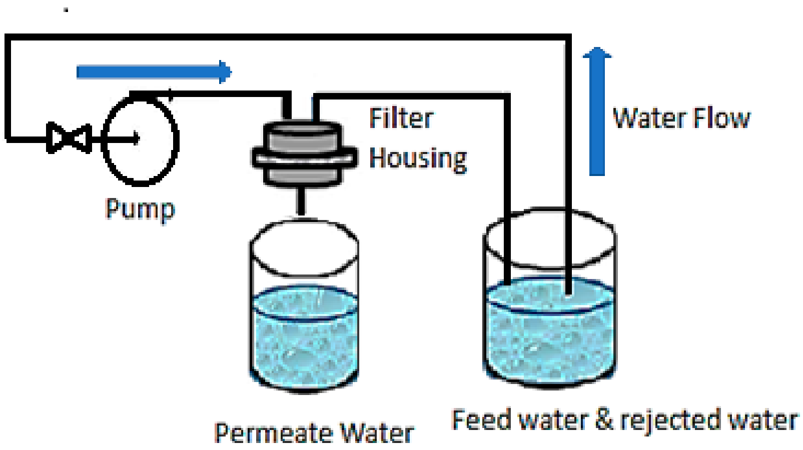

Figure S1. Schematic of filtration system.
